# Editorial: Light manipulation in novel functional materials and waveguide structures

**DOI:** 10.3389/fchem.2022.1054197

**Published:** 2022-10-13

**Authors:** Tingchao He, Min-Cherl Jung

**Affiliations:** ^1^ College of Physics and Optoelectronic Engineering, Shenzhen University, Shenzhen, China; ^2^ Division of Materials Science, Faculty of Pure and Applied Sciences, University of Tsukuba, Ibaraki, Japan

**Keywords:** light manipulation, all-optical polarization manipulation, ferroelectric polarization, band alignment, interlayer coupling, planar fractal microstructure, hot spot

The interaction between light and matter has always been a hot topic in scientific research. One of the important research objectives in the field of optics and photonics is to generate new optical phenomena and obtain optical devices with desired optical functions by the manipulation of the light field. In this Research Topic, we aim to the manipulation of physical properties of novel functional materials, with a focus on the image storage, ferroelectric polarization and hot spot in the dendritic pattern. This Research Topic will allow readers to understand the current progress of light manipulation and reach new ideas for future applications. This research topic includes four original research articles. I are pleased to introduce fascinating results as follows.

Lyu et al. demonstrated the all-optical polarization manipulation through orbital polarization holography in photo-alignment liquid crystals, which was also analyzed theoretically with Jones matrices. This work is promising for the application in light manipulation.

How ferroelectric polarization affects the electronic structure of two-dimensional (2D) materials is rarely investigated. Du et al. found that the band alignments of the In_2_Se_3_/InSe heterostructure can be tuned by the ferroelectric polarization of In_2_Se_3_ and interlayer coupling. This work may provide guidance for the design of novel 2D ferroelectric materials toward the application to integrated optoelectronic devices.

The design of novel micro- and nano-structures will enhance the light–matter interaction and facilitate the rapid development of optoelectronic devices. Kan et al. fabricated the planar fractal microstructure on the silver film treated by positive corona discharge, which exibits the high density of the hot spot in the dendritic pattern and will be used for low-cost SERS detection and fractal photonic metamaterials.

In addition, Yan at al. reported the synthesis of colorized and magnetic polystyrene/Fe_3_O_4_ beads with millimeter size *via in situ* suspension polymerization of styrene.

Finally, we would like to thank all authors, reviewers and administrative staff, without whom this Research Topic could not have been possible.

